# Over-the-counter use of short-acting beta-2 agonists: a systematic review

**DOI:** 10.1186/s40545-023-00627-z

**Published:** 2023-10-09

**Authors:** Zhe Chi Loh, Rabia Hussain, Siew Chin Ong, Bandana Saini, Jaya Muneswarao, Anees ur-Rehman, Zaheer-Ud-Din Babar

**Affiliations:** 1 School of Pharmaceutical Sciences, Universiti Sains Malaysia, 11800 Gelugor, Pulau Pinang Malaysia; 2 Faculty of Medicine and Health, University of Sydney School of Pharmacy, The University of Sydney, Sydney, NSW 2006 Australia; 3 Pharmacy Department, Hospital Pulau Pinang, 10990 George Town, Pulau Pinang Malaysia; 4 Bahauddin Zakariya University, Multan, Pakistan; 5 Department of Pharmacy, University of Huddersfield, Huddersfield, HD1 3DH UK

**Keywords:** Short-acting beta-2 agonists, SABA, Over-the-counter, Prevalence, Factors, Characteristics features

## Abstract

**Background:**

The widespread use of short-acting beta-2 agonists (SABA) as an as-needed treatment for asthma is well-established. However, excessive use of SABA has been linked to undesirable outcomes such as increased risk of asthma attacks, exacerbations, and even death. The availability of SABA as an over-the-counter (OTC) medication has contributed to their overuse, leading to undertreated asthma and reduced access to asthma education.

**Objective:**

This systematic review aimed to summarize the prevalence, characteristic features of, and factors contributing to over-the-counter SABA purchase or overuse.

**Methods:**

The databases searched included PubMed, Scopus, Springer Link, Google Scholar, CINAHL, and APA PsycArticles. Original research articles reporting the prevalence, characteristics features, and factors regarding over-the-counter SABA use, available as full text, published in English language between the year 2000 and April 2023 were included in this review. Commentaries, letters to editor, review articles, qualitative studies, clinical trials, and conference proceedings were excluded. Data extraction was followed by a review of the quality of studies included and data were then synthesized for meaningful findings. This systematic review had been registered in the PROSPERO with registration number CRD42023421007.

**Results:**

A total of 18 articles were included. The prevalence range of OTC SABA users in populations were 1.4% to 39.6% and SABA over-users among OTC users were 14% to 66.4%. Factors mostly associated with this behavior were moderate–severe asthma, and less use of preventers. On top of that, not understanding the risk of SABA overuse was clear in many studies that explored this factor.

**Conclusion:**

Over-the-counter purchase and overuse of SABA medication is a common problem, leading to adverse consequences such as uncontrolled asthma and increased healthcare utilization. Addressing these issues requires improved patient education about their conditions and adequate information regarding the potential long-term effects of SABA use by the healthcare providers. Management and education of asthma patients, including regular monitoring and follow-up, can help reduce overuse of SABA medication and prevent negative consequences.

**Supplementary Information:**

The online version contains supplementary material available at 10.1186/s40545-023-00627-z.

## Background

Short-acting beta-2 agonists (SABAs) are frequently used for symptom relief in asthma on an as-needed basis [1, 2]. Their mechanism of action involves bronchodilation through adrenergic stimulation via the beta-2 receptors on airway smooth muscle cells. The main drugs available in inhalable formulations in this class include salbutamol (albuterol) and terbutaline. Of these, salbutamol is more widely available globally. Previous research has linked the excessive usage of SABAs, particularly when used without anti-inflammatory or preventer medications to adverse consequences such as the occurrence of asthma exacerbations, poorer control of symptoms, use of healthcare resources such as doctor visits or hospital admissions, and even asthma-related mortality [3–7]. The operational definition of SABA overuse in this study refers to the use of three or more SABA canisters in a year [8–11]. Based on robust evidence, the Global Initiative for Asthma Report (GINA, 2023), now recommends that adult patients with asthma (even with mild asthma) use a combination of a SABA and a low dose inhaled corticosteroid in the same or separate inhalers for symptom relief as the first step of asthma treatment [10]. This is a paradigm shift from previous GINA guidelines where SABA use alone was considered the initial treatment for asthma patients with mild symptoms [12, 13].

The overuse of SABA in certain countries may, in part, be due to non-prescription-based accessibility such as Australia, Italy and Spain, where salbutamol inhalers are available as non-prescription medicines [14–16]. Because of this, some patients who believe their symptoms are controllable through use of SABAs alone may rely on these inhalers to manage the condition in these countries [17]. This overuse is also perpetuated by the belief that SABAs provide faster relief, especially when compared to inhaled corticosteroids which do not immediately address symptoms and may take a few weeks for effect [14, 17, 18]. For instance, in Australia, researchers suggest that patients with mild asthma symptoms may choose not to seek medical attention, given they can access over-the-counter salbutamol inhaler relieves for their symptoms [19]. Further, in countries, where salbutamol is available without prescription, it is possible that people may purchase SABAs for respiratory symptoms without being diagnosed with asthma [20].

Additionally, infrequent doctor visits and the over-the-counter purchase of SABA have been connected to undertreated asthma, more severe and frequent symptoms, and reduced access to asthma education [21–23]. SABA use can also mask underlying symptoms, leading to delayed medical help seeking in acute exacerbations [21, 23]. In countries where SABA supply does not require a prescription it has been suggested that to decrease the prevalence of SABA overuse, monitoring, and surveillance of the frequency of purchases of over-the-counter medications are necessary [24]. In such countries where SABA supply does not currently require a prescription, the onus of responsible supply, particularly rests with community pharmacists [25]. Of course, SABA usage may be high even when these are prescription only, as patients may preferentially rely on these rather than on other prescribed preventer medications. However, prescription requirements and the need to have a doctor review for obtaining prescriptions provides some safeguards for patients. Therefore, understanding the contributors to the overuse of over-the-counter SABA is critical for developing effective strategies to combat this issue.

This systematic review aims to identify the prevalence of over-the-counter SABA overuse, user’s characteristic features associated with this overuse, and other systematic factors contributing to the overuse of non-prescription SABA. By summarizing the available evidence, this review seeks to provide a comprehensive understanding of the issue, identify gaps in the literature, and inform future research directions.

## Methods

### Study design

A systematic review was conducted in line with the Preferred Reporting Items for Systematic Reviews and Meta-Analyses (PRISMA) standards [26]. Also, this systematic review was registered in the PROSPERO database (Registration number: CRD42023421007).

### Literature search and search strategy

**Fig. 1:**
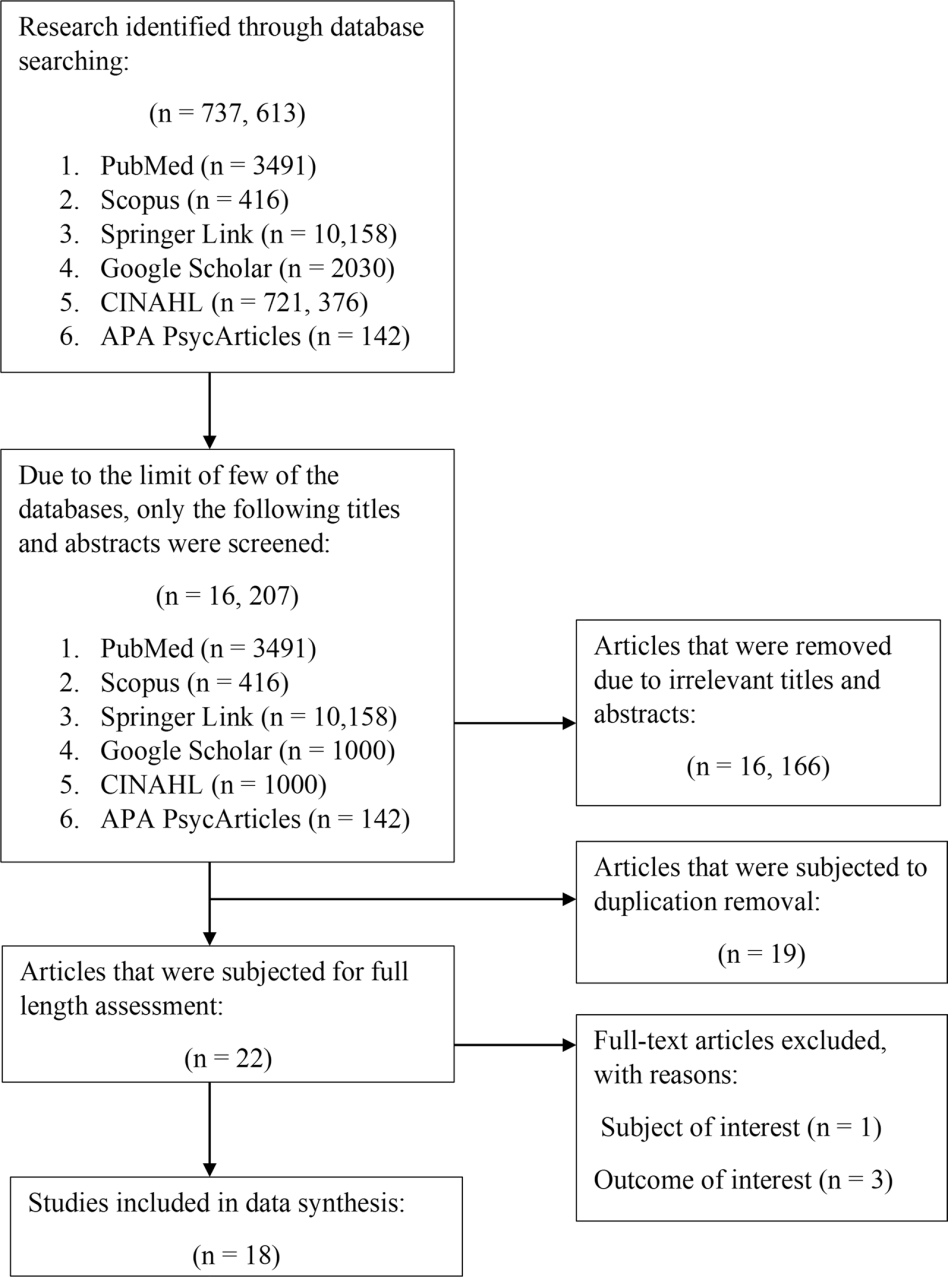
A flow diagram for study selection

Two authors (ZCL and RH) independently carried out an extensive search using various databases such as PubMed, Scopus, Springer Link, Google Scholar, CINAHL, and APA PsycArticles. These databases provide a comprehensive range of biomedical, health science, nursing, and psychology based peer-reviewed articles [27, 28]. The search was limited to original research articles published between the years 2000 and April 2023. Boolean operators “AND” and “OR” were employed to connect the search terms, as shown in Table 1. The search terms were *“overuse”, “misuse”, “excessive use”, “over-the-counter”, “OTC”, “retail”, “community pharmacy”, “without prescriptions”, “non-prescriptions”, “short-acting beta-2 agonists”, “SABA”, “salbutamol”, “terbutaline”, “albuterol”, “asthma reliever”.* The entire process of study selection is shown in Fig. 1. We also undertook a citation chaining exercise by exploring the reference lists of included articles to ensure that no qualifying studies were missed. A consensus was reached between the two authors (ZCL and RH) by engaging in extensive discussion to settle any disagreements on inclusion using a rubric based on the defined inclusion/exclusion criteria.

**Table 1:** Databases and keywords used in search strategies

Database	Search strategy
PubMed	((("overuse"[All Fields] OR "overused"[All Fields] OR "overuser"[All Fields] OR "overusers"[All Fields] OR "overuses"[All Fields] OR "overusing"[All Fields] OR ("misuse"[All Fields] OR "misused"[All Fields] OR "misuser"[All Fields] OR "misusers"[All Fields] OR "misuses"[All Fields] OR "misusing"[All Fields]) OR ("excess"[All Fields] OR "excesses"[All Fields] OR "excessive"[All Fields] OR "excessively"[All Fields])) AND ("nonprescription drugs"[MeSH Terms] OR ("nonprescription"[All Fields] AND "drugs"[All Fields]) OR "nonprescription drugs"[All Fields] OR ("over"[All Fields] AND "counter"[All Fields]) OR "over the counter"[All Fields] OR "OTC"[All Fields] OR ("communal"[All Fields] OR "communalism"[All Fields] OR "communalities"[All Fields] OR "communality"[All Fields] OR "communally"[All Fields] OR "commune"[All Fields] OR "communes"[All Fields] OR "community s"[All Fields] OR "communitys"[All Fields] OR "residence characteristics"[MeSH Terms] OR ("residence"[All Fields] AND "characteristics"[All Fields]) OR "residence characteristics"[All Fields] OR "communities"[All Fields] OR "community"[All Fields]) OR (("retail"[All Fields] OR "retailed"[All Fields] OR "retailer"[All Fields] OR "retailer s"[All Fields] OR "retailers"[All Fields] OR "retailing"[All Fields] OR "retails"[All Fields]) AND ("pharmacie"[All Fields] OR "pharmacies"[MeSH Terms] OR "pharmacies"[All Fields] OR "pharmacy"[MeSH Terms] OR "pharmacy"[All Fields] OR "pharmacy s"[All Fields])) OR ("prescriptions"[MeSH Terms] OR "prescriptions"[All Fields] OR "prescription"[All Fields]) OR "non-prescriptions"[All Fields])) OR (("short-acting"[All Fields] AND "beta-2"[All Fields] AND ("agonist"[All Fields] OR "agonist s"[All Fields] OR "agonistic"[All Fields] OR "agonistically"[All Fields] OR "agonistics"[All Fields] OR "agonists"[MeSH Subheading] OR "agonists"[All Fields])) OR ("3 2 4 azidobenzamidino ethyl 5 hydroxyindole"[Supplementary Concept] OR "3 2 4 azidobenzamidino ethyl 5 hydroxyindole"[All Fields] OR "saba"[All Fields]) OR ("levalbuterol"[MeSH Terms] OR "levalbuterol"[All Fields] OR "albuterol"[All Fields] OR "albuterol"[MeSH Terms] OR "salbutamol"[All Fields]) OR ("terbutaline"[MeSH Terms] OR "terbutaline"[All Fields] OR "terbutalin"[All Fields]) OR ("levalbuterol"[MeSH Terms] OR "levalbuterol"[All Fields] OR "albuterol"[All Fields] OR "albuterol"[MeSH Terms] OR "salbutamol"[All Fields]) OR (("asthma"[MeSH Terms] OR "asthma"[All Fields] OR "asthmas"[All Fields] OR "asthma s"[All Fields]) AND ("reliever"[All Fields] OR "relievers"[All Fields])))) AND ((booksdocs[Filter] OR clinicaltrial[Filter] OR randomizedcontrolledtrial[Filter]) AND (fft[Filter]) AND (2000:2023[pdat])
Scopus	TITLE-ABS-KEY ((overuse OR misuse OR excessive AND use) AND ( over-the-counter OR otc OR community OR retail AND pharmacy OR without AND prescriptions OR non-prescriptions) OR (short-acting AND beta-2 AND agonists OR saba OR salbutamol OR terbutaline OR albuterol OR asthma AND reliever)) AND (LIMIT-TO (PUBYEAR, 2023) OR LIMIT-TO (PUBYEAR, 2022) OR LIMIT-TO (PUBYEAR, 2021) OR LIMIT-TO (PUBYEAR, 2020) OR LIMIT-TO (PUBYEAR, 2019) OR LIMIT-TO (PUBYEAR, 2018) OR LIMIT-TO (PUBYEAR, 2017) OR LIMIT-TO (PUBYEAR, 2016) OR LIMIT-TO (PUBYEAR, 2015) OR LIMIT-TO (PUBYEAR, 2014) OR LIMIT-TO (PUBYEAR, 2013) OR LIMIT-TO (PUBYEAR, 2012) OR LIMIT-TO (PUBYEAR, 2011) OR LIMIT-TO (PUBYEAR, 2010) OR LIMIT-TO (PUBYEAR, 2009) OR LIMIT-TO (PUBYEAR, 2008) OR LIMIT-TO (PUBYEAR, 2007) OR LIMIT-TO (PUBYEAR, 2006) OR LIMIT-TO (PUBYEAR, 2005) OR LIMIT-TO (PUBYEAR, 2004) OR LIMIT-TO (PUBYEAR, 2003) OR LIMIT-TO (PUBYEAR, 2002) OR LIMIT-TO (PUBYEAR, 2001) OR LIMIT-TO (PUBYEAR, 2000)) AND (LIMIT-TO (DOCTYPE, "ar")) AND (LIMIT-TO ( LANGUAGE, "English"))
Springer Link	(Overuse OR misuse OR excessive AND use) AND (over-the-counter OR otc OR community OR retail AND pharmacy OR without AND prescriptions OR non-prescriptions) OR (short-acting AND beta-2 AND agonists OR saba OR salbutamol OR terbutaline OR albuterol OR asthma AND reliever) Within English, Article, 2000–2023
Google Scholar	(Overuse OR misuse OR excessive AND use) AND (over-the-counter OR otc OR community OR retail AND pharmacy OR without AND prescriptions OR non-prescriptions) OR (short-acting AND beta-2 AND agonists OR saba OR salbutamol OR terbutaline OR albuterol OR asthma AND reliever) (Filter: 2000–2023)
CINAHL	(Overuse OR misuse OR excessive AND use) AND (over-the-counter OR otc OR community OR retail AND pharmacy OR without AND prescriptions OR non-prescriptions) OR (short-acting AND beta-2 AND agonists OR saba OR salbutamol OR terbutaline OR albuterol OR asthma AND reliever) (Filter: Online content only (All articles and eBook), Journal article, Publication date from 2000 to 2023)
APA PsycArticles	(Overuse OR misuse OR excessive AND use) AND (over-the-counter OR otc OR community OR retail AND pharmacy OR without AND prescriptions OR non-prescriptions) OR (short-acting AND beta-2 AND agonists OR saba OR salbutamol OR terbutaline OR albuterol OR asthma AND reliever)

### Inclusion and exclusion criteria

The inclusion criteria for studies included in this systematic review were as follows:

1. the study must be original research,
2. must be published in English language,
3. must be available as full-text articles,
4. asthma patients of any age,
5. study focus only non-prescription usage of SABA,
6. study outcome must focus on the use of oral or inhaled SABA that was only supplied over-the-counter, particularly SABA overuse prevalence, factors associated with overuse, and characteristics of over-users.

On the other hand, we have excluded editorials, letters to the editor, review articles, qualitative studies, or conference abstracts from this systematic review. Additionally, we have excluded the study outcome that focused on the prescribed SABA use, or test of SABA efficacy in combined or alone in a clinical trial.

### Quality assessment

We utilized the STROBE assessment tool, which consists of 22 items covering various aspects of a research article, including the title and abstract (item one), introduction (items two and three), methods (items four to 12), results (items 13 to 17), discussion (items 18 to 21), and funding information (item 22). For the scoring process, we subdivided some of the items (such as item five into 5a, 5b, 5c, and 5d), resulting in a total of 32 smaller items with equal weights. Each item received one point if it was mentioned in the appropriate section of the article, and zero points if not. Items that did not apply to a particular article were noted as non-applicable (NA) [29]. A STROBE score of more than 85% was considered 'excellent', 70% to less than 85% was 'good', 50% to less than 70% was 'fair', and less than 50% was 'poor' [30]. Two authors (ZCL and RH) independently evaluated the quality of each included study, while consensus through discussion and negotiation was used to overcome any differences in quality assessment.

### Data extraction

The data were independently extracted by ZCL and RH into a data extraction spreadsheet made in Microsoft Excel version 2306. Extraction of the data included the characteristics of the eligible studies, including country/countries, study design, year of study, study participants, definition of SABA overuse, prevalence of SABA over-the-counter purchase or SABA overuse, characteristic features associated with SABA over-the-counter purchasers or over-users, and factors of over-the-counter SABA purchase or overuse from all the included studies. These data were further organized and grouped based on the main topics that emerged from the studies [31]. When there were variations in the data captured during the extraction process due to reviewer interpretation, we re-read the article and engaged in a joint discussion. This allowed us to achieve the best possible interpretability, as agreed upon by the two researchers. If a consensus could not be reached, a third opinion from SCO was sought.

### Data synthesis

A thematic analysis was conducted to amalgamate the primary findings from the studies included in this research [32]. This involved identifying significant findings that encapsulated the essential meaning of the study results in textual analyses. The resulting key findings were presented by summarizing commonalities among eligible studies as comprehensive themes [33, 34]. The statistical data were included, where available, to indicate the magnitude of the key findings, it was not used in the data synthesis process. As a result, handling of any missing summary statistics was not needed in this systematic review [17].

## Results

After screening 16, 207 titles and abstracts, 16, 166 articles were excluded due to irrelevant titles and abstracts, 19 studies were subjected to duplication removal, and 22 studies underwent full-length assessment. Four studies were excluded because they did not meet the subject (*n* = 1) or outcome of interest criteria (*n* = 3). Finally, 18 studies were included in this systematic review.

The 18 studies reported data from various countries with different income levels based on World Bank classification [9, 15, 35–50]. A total of 11 high-income countries were represented, in data collected from Australia, Chile, Italy, Kuwait, Oman, Saudi Arabia, Singapore, South Africa, South Korea, Taiwan, and The United Arab Emirates. The studies reviewed also included data from ten upper-middle income countries including Argentina, Brazil, China, Columbia, Costa Rica, Malaysia, Mexico, Russia, Thailand, and Turkey. Furthermore, data from low- and middle-income countries were also reported, including data from Egypt, India, Indonesia, Kenya, and the Philippines.

It was noteworthy that the research design of all 18 studies was cross-sectional; Table 2 displays the study characteristics of all included articles. As seen in Additional file 1 Table S1, only three of the included studies were deemed to be of ‘good’ quality [4, 46, 50], with the remaining studies being of "fair" quality [9, 35, 38, 39, 45, 47, 49].

**Table 2:** Characteristics of the studies

References	Authors	Country/countries	Study design	Year of study	Study participants included or focused upon and numbers recruited	Definition of SABA overuse	Prevalence of SABA over-the-counter purchase or overuse		Characteristics features /factors associated with SABA over-the-counter purchasers/over-users	Comments
							OTC purchasers(% of participants surveyed)	SABA canisters purchased or used		
[35]	Alzaabi et al.	The Gulf cluster of Kuwait, Oman, and The United Arab Emirates (UAE)	A multicounty, multicenter, cross-sectional study (SABINA III sub-analysis study)	May to December 2019	**Study targets**: patients aged ≥ 12 years with a confirmed asthma diagnosis, at least 3 annual consults with a HCP, with complete medical records available for ≥ 12 months prior to study visit, and with no other CRD besides asthma. **Number of participants:** 301 patients from health clinics in Kuwait, Oman, and the UAE	Used ≥ 3 SABA canisters per year	13.3% of participants surveyed used OTC SABAs in 12 months prior to the study visit	Among 13.3% SABA over-the-counter purchasers, 52.2% purchased ≥ 3 SABA canisters (12 months prior to the study visit)	All SABA OTC purchasers had moderate-to-severe asthma. 92.5% OTC SABA users had also received SABA prescriptions. Among those patients, 51.4% were prescribed ≥ 3 SABA medications, while 13.5% were prescribed 10 or more SABA medications	This study provides a comprehensive assessment of SABA prescription volumes, SABA OTC purchases, and asthma outcomes in the 3 countries of Kuwait, Oman, and the UAE
[15]	Azzi et al.	Australia	A real-world cross-sectional observational study	October 2017 to October 2018	**Study targets:** individuals ≥ 16 years or older purchasing OTC SABA for themselves from community pharmacies. **Number of participants:** 412 SABA users from 18 community pharmacies in the state of New South Wales, Australia	Used SABA more than twice a week in the previous one month	All patients as study targeted only OTC users	70.1% of participants were defined as over-users based on using their SABA inhalers > twice a week for symptom relief	Compared to non-over-users, a higher % of SABA over-users (59.0%) had uncontrolled asthma, with a ↑ likelihood of using OCS to manage exacerbations (26.2%), visiting the doctor for asthma in past 12 months (74.5%), and having moderate–severe nasal symptoms (80.8%) 73.6% reported not using a preventer daily and only 81.6% reported a doctor diagnosis of asthma	OTC SABA seekers probably have a higher burden of illnesses including rhinitis, need for OCS, frequent health care utilization This study highlights the necessity of involving community pharmacists in directing appropriate use of SABAs in countries where SABA is available OTC
[37]	Bateman et al.	24 countries, i.e., Russia, Australia, South Africa, Kenya, Egypt, Turkey, Saudi Arabia, the United Arab Emirates, Kuwait, Oman, Mexico, Costa Rica, Columbia, Chile, Brazil, Argentina, Thailand, Taiwan, South Korea, Singapore, Philippines, Malaysia, Indonesia, and India	A multi-country, observational, cross-sectional study (SABINA III main study)	March 2019 to January 2020	**Study targets:** patients ≥ 12 years, with documented asthma diagnosis in their medical records, ≥ 3 prior consults with HCP /year complete medical records for ≥ 12 months prior to the study visit, and no other CRD except asthma. **Number of participants:** 8,462 patients recruited from primary and specialist healthcare settings in 24 countries	Used ≥ 3 SABA canisters per year	18.0% of included participants were OTC SABA purchasers (12 months prior to the study visit)	48.8% of participants purchased ≥ 3 SABA canisters (12 months prior to the study visit)	Among those purchasing non -prescription SABA, 76.8% had also received SABA prescriptions, and 69.9% obtained ≥ 3 canisters and 35.8% obtained ≥ 10 canisters in the past 12 months using prescriptions	SABA OTC purchases need to be regulated and automatic repeat prescriptions may result in high and unnecessary prescriptions
[38]	Yorgancıoğlu et al.	Turkey	An observational, cross-sectional study (SABINA III sub-analysis study)	September 2019 to January 2020	**Study targets:** patients aged ≥ 12 years with a documented asthma diagnosis of asthma, ≥ three consults with a HCP and available complete medical records for ≥ 12 months prior to with no CRD except asthma. **Number of participants:** 588 patients recruited from 24 centers in Turkey	Used ≥ 3 SABA canisters per year	10.2% of participants were OTC SABA users (12 months prior to the study visit)	27.1% of them purchased ≥ 3 SABA canisters (12 months prior to the study visit)	A higher proportion of patients with moderate-to-severe asthma than those with mild asthma purchased ≥ 3 SABA canisters without a prescription (29.4% vs 12.5%)	There is a non-representative asthma study population from specialist care, leading to a greater number of moderate-to-severe asthma patients being included in the study
[39]	Al-Jahdali et al.	Saudi Arabia	An observational, cross-sectional, cohort study (SABINA III sub-analysis study)	March 2019 and January 2020	**Study targets:** patients ≥ 12 years, with a documented asthma diagnosis of asthma, with at least 3 consults with HCP and complete medical records of these for past 12 months and with no other CRD except asthma. **Number of participants:** 502 patients recruited by pulmonologists or respiratory medicine physicians	Used ≥ 3 SABA canisters usage per year	21.9% participants in the 12 months prior to the study visit had used OTC SABAs	66.4% of participants had purchased ≥ 3 SABA canisters in 12 months prior to the study visit	Among patients who purchased ≥ 3 SABA canisters, 69.3% of patients experienced moderate-to-severe asthma and 33.3% had milder asthma. Most patients who purchased SABA over-the-counter also received SABA prescriptions (88.2%), and among these, 80.4% received ≥ 3 SABA prescriptions and 56.7% received ≥ 10 SABA prescriptions over the last 12 months	This study lends weight to the fact that prescribing SABAs in asthma may signal to patients that these are safe and catalyze OTC use (which then may or may not be disclosed other to HCPs) Again, the role of pharmacists in instigating three way communication with patients and their doctors around SABA overuse is notably important
[40]	Douglass et al.	Australia	A real-world cross-sectional observational study	June 2004 and July 2005	**Study targets:** individuals aged ≥ 16 years who purchased SABA medications for personal use recruited by community pharmacists. **Number of participants:** 316 asthma patients from 43 metropolitan and regional community pharmacies	Not stated, it may be noted that GINA definition of overuse was published later than this study	39.6% of participants surveyed used SABAs OTC	Not stated	45% of participants had impaired lung function with a forced expiratory volume (FEV1) less than 80% predicted, 34.7% of these purchased their SABA without a prescription. Moreover, individuals who purchased their SABA with a prescription were more likely to have used preventer medication in the past seven days	It appears a third of OTC SABA purchasers had less than normal healthy lung function. Prescription SABA use was associated with preventer use It is likely that this situation may have been worse in regional -rural areas where HCP shortages are more likely – this study was metropolitan area based
[41]	Bao et al.	China	A multicenter, observational, cross-sectional study (SABINA III sub-analysis study)	March and August 2020	**Study targets:** patients ≥ 12 years with a documented asthma diagnosis confirmed by the presence of recurrent symptoms and spirometry with, at least 3 annual consultations with HCP, and no other CRD other than asthma. **Number of participants:** 498 patients recruited from 25 tertiary centers (specialists) across China	Used ⩾ 3 SABA canisters per year	5.2% were OTC SABA purchasers in the 12 months prior to the study visit	50% of participants purchased ⩾ 3 SABA canisters (12 months prior to the study visit)	Not stated	This study was to assess the treatment patterns and clinical outcomes in patients with predominantly moderate-to-severe asthma, who were managed at tertiary centers at China
[36]	Azzi et al.	Australia	A real-world cross-sectional observational study	October 2017 and October 2018	**Study targets:** SABA users who were aged 16 years or older, purchased over-the-counter SABA medication for themselves from community pharmacy, and were able to communicate in English. **Number of participants:** a total of 412 participants were recruited from 18 different community pharmacies	Used SABA > twice in the past one month	Not applicable	Not stated	Based on GINA-defined criteria, 47% of the participants had uncontrolled asthma in the previous 4 weeks. Recent high SABA users were found to be more likely to have uncontrolled asthma compared to recent non-high SABA users. Additionally, a significantly higher proportion of high SABA users agreed that asthma impacted their daily activities, aware of the social implications of their condition in comparison to non-high SABA users	Of the participants who considered their asthma to be completely or well-controlled, more than half (60.5%) reported overused SABA. A statistically significant proportion of high SABA users (49.8%) reported the need for additional information compared to non-high SABA users (27.6%). Furthermore, high SABA users (46.2%) were more likely to rate their asthma as somewhat controlled when compared to non-high SABA users (11.2%)
[42]	Montero-Arias et al.	Argentina, Brazil, Chile, Colombia, Costa Rica, and Mexico	A cross-sectional, multicounty, multicenter, observational study (SABINA III sub-analysis study)	Not stated	**Study targets:** patients with asthma aged ≥ 12 years with ≥ 3 HCP consults and complete medical records for ≥ 12 months before the study visit, and no other CRD other than asthma. **Number of participants:** 1096 patients, recruited by primary care physician and specialists from medical settings in Argentina, Columbia, and Mexico	Used ≥ 3 SABA canisters per year	17.2% of participants purchased SABAs OTC in the 12 months prior to the study visit	38.8% and 7.4% purchased ≥ three and ≥ 10 canisters of SABA, respectively, in the 12 months prior to the study visit	Of the patients who purchased SABA over-the-counter, 80.3% were also prescribed SABA, either as monotherapy or in addition to maintenance therapy. Within the preceding 12 months, 73.5% were prescribed ≥ 3 SABA canisters, with 53.6% prescribed ≥ 10. Among the patients who overused SABA, 22.7% believed the medication to be safe to use. About half (49.5%) high SABA users expressed concern about the potential long-term effects of using inhaled corticosteroids, in comparison to non-high SABA users SABA overuse appeared more prevalent in primary versus specialist care patients (29.2% vs 15.6%, respectively)	Higher SABA users may be resorting to SABs as nearly half had concerns about inhaled steroid preventers while a fifth believed that SABAs were safe. Education that helps patients accurately weigh benefit versus risks of both SABAs and steroid preventers may be useful in diluting SABA overuse It was clear that
[43]	Avdeev et al.	Russia	A multi-country, cross-sectional observational study (SABINA III sub-analysis)	March 2019 to March 2020	**Study targets:** participants aged 12 years or older, who had a diagnosis of asthma and had undergone at least 3 consultations with healthcare professionals with medical records available for at least 12 months prior to the study visit, and no other CRD other than asthma were recruited from 12 Russian centers. **Number of participants:** a total of 618 participants were included in the final analysis of the study	Used ≥ 3 SABA canisters per year	30.1% (12 months prior to the study visit)	14% purchased ≥ three canisters of SABA per year over-the-counter (12 months prior to the study visit)	The quantity of SABA purchased without a prescription was generally not associated with asthma severity. However, among those with mild asthma, 7% purchased ≥ 6 SABA canisters per year, which was a slightly higher percentage than those with moderate/severe asthma, of whom only 5% purchased six or more canisters annually. Of those who acquired SABA over-the-counter, 91% already received SABA prescriptions and 59% received prescriptions for three or more SABA canisters in the past year	Not stated
[44]	Price et al.	24 countries which include Russia, Australia, South Africa, Kenya, Egypt, Turkey, Saudi Arabia, the United Arab Emirates, Kuwait, Oman, Mexico, Costa Rica, Columbia, Chile, Brazil, Argentina, Thailand, Taiwan, South Korea, Singapore, Philippines, Malaysia, Indonesia, and India	A multi-country, cross-sectional observational study (SABINA III sub-analysis study)	Not stated	**Study targets:** patients with asthma, aged 12 years or older, who had undergone at least three consultations with healthcare professionals, had medical records with at least 12 months of data prior to the study visit, and no other chronic respiratory disease other than asthma were recruited by primary care physicians and specialists from 24 countries across five continents. **Number of participants:** a total of 8351 participants were included in this study	Used ≥ 3 SABA canisters per year	21.5% (12 months prior to the study visit)	Not stated	Mild asthma patients preferred to purchase SABA over-the-counter than moderate-to-severe asthma (24.1% vs 18.4%). Among patients with both SABA over-the-counter purchase and prescriptions, most had already been prescribed ≥ three (72.7%) and ≥ 10 (39.1%) canisters	Not stated
[45]	Reddel et al.	Australia	A cross-sectional web-based survey	Not stated	**Study targets:** participants with current asthma, aged 16 years or older and residing in Australia, were recruited by Survey Sampling International (Melbourne, Australia) with the help of 224,898 panels. **Number of participants:** a total of 2686 participants were included in this study.	Not stated	24.3%	Not stated	Participants who only used relievers and purchased them over-the-counter had similar levels of symptom control compared to those who primarily purchased relievers using prescriptions, and there was little difference in urgent healthcare use. However, individuals who purchased their relievers over-the-counter were less likely to have visited a general practitioner for a non-urgent asthma review (27.0%) when compared to those who primarily used prescription-based relievers (44.0%)	Participants who had a medication concession card were significantly less likely to purchase their relievers over-the-counter compared to those without a concession card (20.8% versus 52.3%). In addition, individuals who only used relievers were more likely to purchase their relievers over-the-counter than those who used preventers
[9]	Marco et al.	Italy	A cross-sectional and retrospective study (SABINA II sub-analysis study)	June and July 2019	**Study targets:** asthma patients who purchased SABA inhalers from retail pharmacies **Number of participants:** data was collected from 1136 subjects using pop-up surveys across 200 Italian retail pharmacies.	Used ≥ 2 SABA canisters per year	15%	Not stated	Not stated	Not stated
[46]	Campbell et al.	Australia	A cross-sectional study	1993	**Study targets: ** asthma patients who purchased SABA inhalers from eight community pharmacies in Adelaide were recruited. **Number of participants:** a total of 129 participants were included in the study	Not stated	Not stated	Not stated	9% of those who purchased SABA over-the-counter had been admitted to hospitals. 60% of purchasers reported that they have symptoms such as cough, wheeze, or shortness of breath, at least every day or night	Many people do not see much value in visiting a doctor only to obtain a prescription. They often have experiences where the only thing offered during consultations is a script for Ventolin, without any further discussion or review of their condition. Patients have expressed a desire for more information and to have more discussions with their general practitioners. Some people feel that their general practitioners should listen more to their concerns about asthma Some patients choose to purchase SABA medications over-the-counter due to convenience, such as being too busy or living too far from healthcare settings. For others, visiting a doctor would result in additional charges, acting as another barrier to their decision. Some patients prefer to have personal control over their asthma by using SABA medications, as Ventolin is perceived to be an instantly effective medication for relieving symptoms. This sense of personal control is further facilitated and reinforced by using Ventolin
[47]	Shen et al.	Taiwan	An observational, cross-sectional s (SABINA III sub-analysis study)	June 2019 to December 2019	**Study targets:** patients who were aged 18 years or older, had a documented diagnosis of asthma, had undergone at least three healthcare provider consultations, had medical records with at least 12 months of data prior to the study visit, were eligible for enrollment, and no other chronic respiratory disease other than asthma. **Number of participants:** a total of 294 patients who met these criteria and were recruited from eight sites in Taiwan were included in the final analysis	Used ≥ three SABA canisters per year	1.4%	0.7% purchased one to two SABA canisters, 0.7% purchased ≥ three SABA canisters (12 months prior to the study visit)	Not stated	Not stated
[48]	Pedrozo-Pupo et al.	Columbia	An observational, cross-sectional study (SABINA III sub-analysis study)	August 2019 to December 2019	**Study targets:** patients aged 12 years or older with a documented diagnosis of asthma, who had undergone at least three consultations with a healthcare professionals or practice and had medical records with at least 12 months of data prior to the study visit, and no other chronic respiratory disease other than asthma were enrolled. **Number of participants:** a total of 250 patients who met these criteria and were recruited from seven medical centers across Columbia were included in the study	Used ≥ 3 SABA canisters per year	17.6%	43.2% purchased ≥ three SABA canisters (12 months prior to the study visit)	Not stated	Not stated
[49]	Modi et al.	India	A multicenter, observational, and cross-sectional study (SABINA III sub-analysis study)	August to December 2019	**Study targets:** adult and adolescent patients (aged ≥ 12 years) with a documented diagnosis of asthma and ≥ 3 consultations with the same healthcare practitioners during the 12 months preceding the study visit were included in the study. **Number of participants:** a total of 510 patients were included from 12 sites of India	Used ≥ 3 SABA canisters per year	8.0%	Among the 41 patients who purchased SABA over-the-counter, 38.9% (n = 16) had purchased ≥ three canisters in the 12 months preceding the study visit	Not stated	Not stated
[50]	Khattab et al.	Africa (Egypt, South Africa, and Kenya	A cross-sectional, multi-country, multi-center observational study (SABINA III sub-study)	Not stated	**Study targets:** patients aged ≥ 12 years, with a documented physician diagnosis of asthma in their medical records, ≥ 3 prior consultations with their healthcare professionals, and medical records containing data for ≥ 12 months before the study visit. **Number of participants:** a total of 1778 patients were included in the final analysis (49%, 28.2% and 22.8% of patients were recruited in Egypt, South Africa, and Kenya, respectively. Data of African cohort were exported using electronic case report forms	Used ≥ 3 SABA canisters per year	32.6%	51.8% and 6.0% purchased ≥ three and ≥ 10 SABA canisters, respectively (12 months prior to the study visit)	Of patients with both SABA over-the-counter purchase and SABA prescriptions, 71.9% had received prescriptions for ≥ three SABA canisters and 40.1% had received prescriptions for ≥ 10 SABA canisters in the previous 12 months. A higher proportion of patients treated in primary care had SABA over-the-counter purchases compared to those treated in specialist care (36.8 vs 29.6%)	Not stated

*CRD* chronic respiratory disease

*HCP* healthcare professionals

*OCS* oral corticosteroids

*OTC* over-the-counter

*SABA* short-acting beta-2 agonists

The findings of this systematic review were categorized into three main themes:

1. Prevalence of SABA over-the-counter purchase or overuse,
2. Characteristic features associated with SABA over-the-counter purchasers or over-users,
3. Factors potentially contributing to over-the-counter SABA purchase or overuse.

### Prevalence of SABA over-the-counter (OTC) purchase or overuse

OTC SABA use ranged between 1.4% to 39.6% as shown in Table 2. Australia recorded the highest prevalence (39.6% in 2005) of SABA over-the-counter purchasers among the asthma population studies [40]. The second highest prevalence was reported in Egypt, South Africa, and Kenya, with 32.6% of patients purchasing SABA over-the-counter [50]. In 2020, Russia reported 30.1% of the surveyed asthma population purchasing SABA over-the-counter [43]. In Australia, over-the-counter SABA overuse showed a decreasing trend between the year 2005 to 2017, with 39.6% and 24.3% of study participants reporting over-the-counter SABA use, respectively [40, 45]. Further, in the Australian context, it was evident that 70.1% to 73.9% of people with asthma participating in the studies who purchased SABA over-the-counter at community pharmacies were SABA over-users, meaning they used SABA more than twice a week in the past 4 weeks [15, 36]. Our polled data for all studies reviewed indicate that 14% to 66.4% of asthma patients had purchased three or more SABA canisters over-the-counter over a period of 12 months [35, 37–39, 41–43].

### Characteristic features associated with SABA over-the-counter purchasers or over-users

The studies reviewed revealed varying preferences for over-the-counter SABA purchases among patients with both moderate-to-severe and mild asthma [38, 39, 43, 44]. One-third of patients in Turkey (29.4%) and two-thirds of patients in Saudi Arabia (69.3%) preferred to purchase SABA without a prescription while having moderate-to-severe asthma [38, 39]. In the same studies, in contrast, only 12.5% and 33.3% of patients with mild asthma in Turkey and Saudi Arabia, respectively, made over-the-counter purchases [38, 39]. Conversely, two other studies reported that mild asthma patients were more likely to purchase over-the-counter SABA (7% and 24.1% in these studies) compared to those with moderate-to-severe asthma (5% and 18.4%) [43, 44]. Further, most of the studies reviewed indicated that most over-the-counter SABA users also had SABA prescriptions, that 29.4% to 80.4% had received three or more SABA prescriptions and 13.5% to 56.7% had been prescribed with 10 or more SABA canisters in the past one year [35, 37–39, 42, 44].

Over-the-counter SABA users or over-users were more likely to have uncontrolled asthma, experience worsening symptoms that required oral corticosteroids, visit the doctor more frequently for asthma, and have moderate-to-severe nasal symptoms [15]. Additionally, recent high SABA users were more likely to have uncontrolled asthma, which significantly impacted their daily activities and social life compared to non-high SABA users [36].

### Factors contributing to SABA over-the-counter purchases or overuses

The over-the-counter purchase of SABA in the reviewed studies was linked to various factors including patients’ perceived control over the asthma, the level of information about their conditions, choice of healthcare providers, level of medication affordability, and personal preferences [36, 42, 45, 46]. For instance, one of the studies claimed that patients receiving care from primary health care professionals were more likely to purchase SABA medicines over-the-counter compared to those receiving specialist/tertiary care [42]. In another study, patients who held a concessional status for lowered medicine co-payments on prescriptions tended to favor prescriptions rather than over-the-counter SABA purchase in countries with publicly funded healthcare systems [45]. It may also be the case that patients who choose to purchase SABA medication over-the-counter may do so either for convenience or personal control over their asthma [46].

Overuse of SABA medication may occur due to concerns about the safety of inhaled corticosteroids and a lack of awareness regarding the potential long-term effects of SABA use [42] Notably, individuals who only used relievers were more likely to purchase them over-the-counter than those who used preventers [45]. Many patients desired more information and discussions with their general practitioners about their condition, and some believed that their healthcare providers should be more attentive to their asthma-related concerns [46].

Misperceptions about one’s own asthma control were also a contributor to over-the-counter SABA use or overuse. Interestingly as depicted by Azzi et al. (2022), a significant proportion of patients with asthma, who considered their asthma to be well or completely controlled, overused SABA medication, leading to suboptimal control of their asthma [36]. High SABA users appeared to require more information about their condition, and they rate their asthma as somewhat controlled more often than non-high SABA users [36].

## Discussion

This study is the first such review pooling data on the prevalence of over-the-counter SABA purchase and overuse, user’s characteristic features associated with this phenomenon or the factors contributing to the overuse of non-prescription SABA globally. The review findings demonstrate that the over-the-counter purchase of SABA is a prevalent phenomenon in several countries, and a significant percentage of patients who purchased SABAs without a prescription overused them [9, 15, 35–45]. Much of this data has emerged in the recent few years, implying the relevance of the issue and the need to act now to handle this issue at a global level through specific systemic level interventions.

In many studies that were included in the review, SABA overuse was associated with underuse of preventers [51, 52]. The results of several studies included in this review indicated that asthma patients may tend to over-rely on SABA due to inability to cope with the situation, social challenges, fear of symptoms, and the belief that they cannot manage their condition without SABA inhalers [1, 17, 53, 54]. These results match expert opinion, or results of research not included in this review (i.e., studies that did not capture prevalence of SABA OTC use or overuse) [50, 51]. This behavior is quite likely as patients mainly seek immediate relief of symptoms rather than managing the underlying condition. SABAs provide immediate relief that is perceivable, while inhaled corticosteroids do not have an immediate perceivable effect. Experts highlight that initial treatment using SABA monotherapy in mild cases where patients experience infrequent symptoms may signal to them that SABAs are safe and effective [54].

Although this may be a bidirectional issue, the findings of our review also suggest that the over-the-counter overuse of SABA medications are likely associated with negative consequences such as uncontrolled asthma, worsening symptoms that necessitate the use of oral corticosteroids, frequent doctor visits for asthma, and moderate-to-severe nasal symptoms, especially among patients with moderate-to-severe asthma [15, 35, 36, 38–40, 43–46]. Another study has shown that the integration of a structured evidence-based pharmacist-delivered service at community pharmacies helped patients better manage their asthma, allergic rhinitis, use of relievers, and health care utilization [55].

In our review, Australia, Egypt, Kenya, Russia, and South Africa, were countries with a high prevalence of over-the-counter use and potential overuse of SABA medications [40, 43, 50]. In these countries, SABA medications are available in community pharmacies without a prescription. This may be a systems-based decision based on asthma prevalence, as asthma appears to be a prevalent respiratory condition in these countries, affecting a significant proportion of the population. For instance, around 11% of Australians have a diagnosis of asthma, while 6.7% and 7.5% of Egyptians and Kenyans, respectively, have been reported to have the condition [56–58]. Similarly, current asthma prevalence among South African adults was reported to be 21.3%, and the adult asthma prevalence ranged from 7.4% to 10.6% in Russia [59–61]. Given the high burden of asthma in these countries, there is a possibility that many people may require quick and easy access to SABA medications to manage their symptoms. The high occurrence of SABA over-the-counter purchases in Russia, with online medicine sales accounting for 7.5% of total pharmacy sales in 2021 and 12% of Russians ordering from online pharmacies, further substantiates the importance of convenient access of these medications for individuals with asthma in countries where the condition was prevalent [62, 63].

The studies have revealed that the overuse of over-the-counter medicines is a global issue, and that the frequent use of such medications can make those with asthma more vulnerable to adverse drug outcomes [64–66]. Community pharmacists are well-positioned to play a role in medication review for this patient population, as studies have shown that pharmacist-led medication review can successfully improve health outcomes for patients with chronic conditions such as asthma [67]. One potential way to curtail this SABA overreliance may be to have regulated oversight of SABA purchases made over-the-counter to reduce the patients’ excessive reliance on SABA medicines or to have campaigns that are thoughtfully designed and use key principles of risk communication targeting those with asthma. Indeed, Gray and Press (2022) suggested that salbutamol should not be available for over-the-counter purchases. However, this may curtail access to the medication in emergency situations such as extreme weather conditions (bushfires, thunderstorm asthma) or even pandemic based lockdowns and closure of primary care facilities Indeed, Feldman et al. (2022), argued that making SABA medications available over-the-counter could save millions of uninsured citizens in the United States, as the cost of these medications, and the issue of poor inhaler technique should be addressed by professional societies, rather than by restricting access to over-the-counter medicines [68]. In Australia a regulatory strategy has been implemented to schedule SABAs as pharmacist only medicines, which places the imperative on pharmacists to review requests for SABAs, review asthma management and provide referrals to doctors for patients where they deem SABA overuse is occurring signaling and uncontrolled asthma [69]. Despite this, SABA overuse in Australia is prevalent and pharmacists may need highly expert risk communication skills to be able to relay the messaging about SABAs effectively [69].

As most patients cannot use respiratory inhalers with the required techniques, thus not getting immediate relief and end up using more doses than intended due to their inappropriate technique of using inhalers [70]. Pharmacists also need to be able to address this issue and should provide a physical demonstration of the correct use of asthma inhalers while dispensing these medications. This could help to ensure that patients use the inhaler correctly and avoid potential adverse outcomes and may help reduce doses used [71]. Moreover, community pharmacists can also play a role in counseling or promoting the appropriate use of preventers for asthma patients [72].

A recent cluster randomized controlled trial undertaken with less poorly controlled asthma patients in community pharmacies, demonstrated that trained community pharmacists were able to shift the reliever-to-preventer use ratio towards a heavier use of preventers. This was the result of a service designed to help ensure guideline concordant use of medicines, improve adherence to prescribed therapy and inhaler use technique, as well as refer to physicians on as-needed basis [73]. This study targeted patients overusing SABAs (prescription or non-prescription) and with poorly controlled asthma measured using a validated questionnaire. While our review focused on OTC SABA use/overuse, there are research studies that have reported no significant differences in clinical outcomes between patients who purchased over-the-counter SABA and those who used prescribed inhalers [40, 68]. For example, Reddel et al. (2017) observed that patients who purchased relievers over-the-counter (35.9%) and those who predominantly used prescriptions (40.6%) had similar levels of symptom control [45]. Also, the fact that many patients who obtain SABA medications without a prescription have also received SABA prescriptions, was highlighted in this review [35, 37, 39, 42–44]. It may be assumed that due to lost or empty inhalers, patients may resort to purchasing over-the-counter medication instead of waiting for a prescription refill. Pharmacists, again have a primary role in these situations, by functioning as a ‘triage’ professional and providing physicians with relevant information after reviewing a patient’s current medicine use (including complementary medicines, level of adherence to prescriptions and other over-the-counter medicine purchases) to prevent treatment overlaps [74].

While most asthma management guidelines globally recommend regular review of asthma, this may not always occur and incentives to health professional end may be required to encourage their safe use of SABA practices. In this review it was evident that a significant proportion of individuals who overuse SABA medication with over-the-counter purchase, perceived their asthma to be well-controlled [36]. This may be since these patients who purchased SABA without prescriptions did not regularly follow up with their physicians and therefore did not receive accurate and up-to-date information about their condition [35, 45, 75]. Sandelowsky et al. (2022) reported that asthma patients who regularly visit primary and secondary care have higher adherence to inhaled corticosteroids [76]. All healthcare providers play a major role in managing asthma patients who purchase SABA medication over-the-counter. This could include a discussion of the need for healthcare providers to communicate with patients and to be aware of what medications the patient has been prescribed with or is currently taking, as well as the need for healthcare providers to listen to patients' concerns about their asthma and to provide education and support [77].

**Fig. 2:**
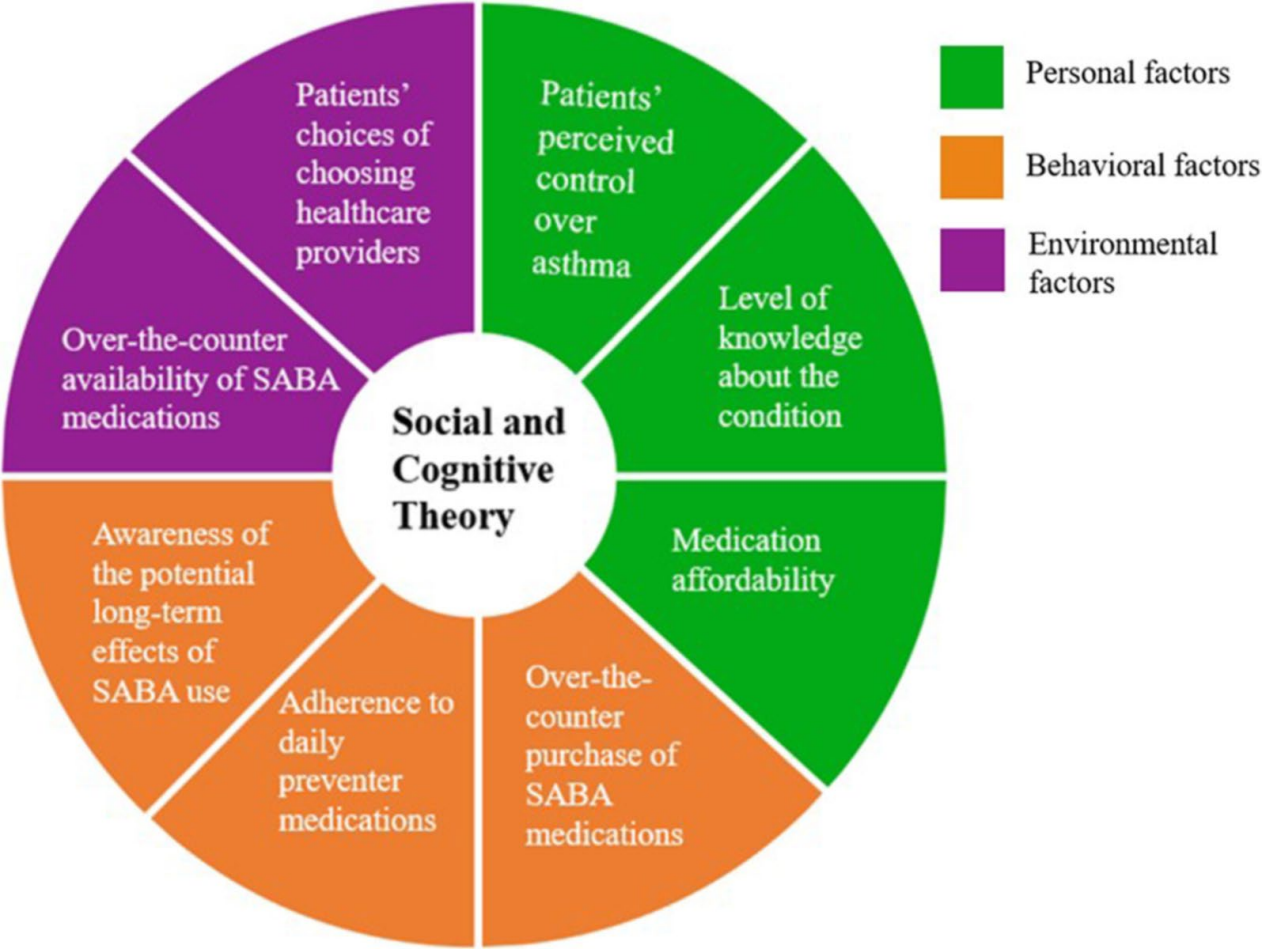
The social and cognitive theory framework diagram

The SCT posits a reciprocal dynamism in that human behavior is influenced by two-way interactions between personal, environmental, and behavioral factors [78, 79]. Personal factors can refer to cognitive, motivational, emotional, demographic, and other identifiable aspects of an individual, such as patients’ perceived control over asthma, level of knowledge about the condition, and medication affordability [80]. Environmental factors may include social and physical factors that affect behavior, such as the over-the-counter availability of SABA medication and patients’ choice of choosing healthcare providers [81]. Behavioral factors, on the other hand, relate to external factors that influence an individual's behavior, as people actively engage with their environment and make choices based on perceived consequences [82]. Social and cognitive theory (SCT) and frameworks can well explain the collated findings of all reviewed studies regarding over-the-counter use of SABA medications in this review. As cited in some of the studies, a prescription at milder symptom stages signaled to patients that SABAs were safe, i.e., it served as an observational learning. Similarly, immediate relief and feeling of improvement after initial SABA use may build expectations that SABAs are safe and effective. Thus, initiating preventers early or alongside SABAs in even mild cases in adult asthma as per the revised GINA guidelines makes good sense from a behavioral perspective also. From this review, it was also clear that there are opportunities to enhance the behavioral capability of patients through education and information provision as patients appeared to misperceive the level of risks associated with SABA overuse (underperceive) and inhaled corticosteroid preventers (over sensitive). Information through public campaigns or individualized education by health professionals can enhance the behavioral capability of patients in making a balanced choice between overusing SABAs and using preventers as prescribed and having their asthma proactively reviewed regularly. Motivation reinforcement when patients attempt to make a balanced decision, can help cement behavior change. By understanding these social cognitive factors, effective behavior change interventions can be developed to promote optimal medication use and improve asthma outcomes (Fig. 2).

Pharmacists, as accessible healthcare professionals, should play a pivotal role in patient education and proper inhaler techniques, empowering individuals to understand and adhere to asthma management plans [83, 84]. Simultaneously, health authorities are tasked with establishing evidence-based guidelines, supporting educational initiatives, and fostering collaboration among healthcare providers [17]. By actively practicing these measures, pharmacists and health authorities can contribute to improved patient outcomes, reduced healthcare burdens, and enhanced overall asthma care, aligning with the principles of evidence-based and patient-centered healthcare [85].

## Strengths and limitations of the review

Although this systematic review offers valuable insights into the use of over-the-counter SABA medicines, it is important to acknowledge its limitations. Such as, many studies may have focused on overuse without differentiating SABA source and had to be excluded leading to a more limited study set for review. Statistical compilation of SABA overuse prevalence was limited by slightly variable definitions of SABA overuse and therefore not undertaken. Most studies that were reviewed focused on overuse prevalence and relating this to potential association with social/asthma demography. Also, not many studies systematically used theoretical frameworks in mapping factors impacting SABA overuse or patient behavior. There were no studies that trialed an intervention specifically to reduce SABA use in a population, hence we could not build effect sizes for these interventions. The article selection was undertaken by two members working in a mentor–mentee team and may therefore have some selection bias. We selected articles in English only, and this may have excluded research published in other languages. Our search strategy also appeared to have picked only a specific set of studies (cross-sectional surveys mainly) and thus may have been skewed to favor this study type.

Despite these limitations, the systematic review provides a comprehensive analysis of the use of SABA medications that are available without a prescription. A notable strength of this study is that it represents the first review specifically centered on non-prescription SABA usage.

## Conclusion

Based on this systematic review, over-the-counter purchase of SABA is common in many countries, and patients who buy them without a prescription tend to overuse them. This issue is associated with negative consequences, such as uncontrolled asthma, more frequent doctor visits, and hospitalizations. Factors influencing the over-the-counter purchase of SABA included receipt of SABA prescriptions, perceived control over asthma, level of information about the condition, choice of healthcare provider, and medication affordability. To address these issues, patients who purchase SABA over-the-counter need more information about their condition, and healthcare providers should provide adequate information about the potential long-term effects of SABA use. Management and education of patients with asthma, including regular monitoring and follow-up, can help reduce overuse of SABA medicines and can prevent negative consequences.

### Supplementary Information

**Additional file 1. **Quality assessment of the included studies.

## Data Availability

All data generated or analyzed during this study are included in this published article and its supplementary information files.

## Abbreviations

SABA: Short-acting beta-2 agonists
CRD: Chronic respiratory disease
HCP: Healthcare professionals
OCS: Oral corticosteroids
OTC: Over-the-counter
SCT: Social and cognitive theory
